# Integrative Analysis of Metabolomics and Transcriptomics Reveals Molecular Mechanisms of Anthocyanin Metabolism in the Zikui Tea Plant (*Camellia sinensis* cv. Zikui)

**DOI:** 10.3390/ijms23094780

**Published:** 2022-04-26

**Authors:** Ju Cai, Litang Lv, Xiaofang Zeng, Fen Zhang, Yulu Chen, Weili Tian, Jianrong Li, Xiangyang Li, Yan Li

**Affiliations:** 1 The Key Laboratory of Plant Resources Conservation and Germplasm Innovation in Mountainous Region (Ministry of Education), College of Life Sciences/Institute of Agro-Bioengineering, Guizhou University, Guiyang 550025, China; jc1294885477@163.com (J.C.); xfzeng1@gzu.edu.cn (X.Z.); 17852027398@163.com (F.Z.); chenyulu295567@163.com (Y.C.); t1321718277@163.com (W.T.); jrli@gzu.edu.cn (J.L.); 2 College of Tea Sciences, Guizhou University, Guiyang 550025, China; ltlv@gzu.edu.cn; 3 State Key Laboratory Breeding Base of Green Pesticide and Agricultural Bioengineering, Key Laboratory of Green Pesticide and Agricultural Bioengineering, Ministry of Education, Guizhou University, Guiyang 550025, China

**Keywords:** anthocyanin metabolism, association analysis, metabolomics, purple-leaf tea plant, transcriptomics, ‘Zikui’

## Abstract

In this study, we performed an association analysis of metabolomics and transcriptomics to reveal the anthocyanin biosynthesis mechanism in a new purple-leaf tea cultivar Zikui (*Camellia sinensis* cv. Zikui) (ZK). Three glycosylated anthocyanins were identified, including petunidin 3-*O*-glucoside, cyanidin 3-*O*-galactoside, and cyanidin 3-*O*-glucoside, and their contents were the highest in ZK leaves at 15 days. This is the first report on petunidin 3-*O*-glucoside in purple-leaf tea. Integrated analysis of the transcriptome and metabolome identified eleven dependent transcription factors, among which *CsMYB90* had strong correlations with petunidin 3-*O*-glucoside, cyanidin 3-*O*-galactoside, and cyanidin 3-*O*-glucoside (PCC > 0.8). Furthermore, we also identified key correlated structural genes, including two positively correlated *F3’H* (flavonoid-3′-hydroxylase) genes, two positively correlated *ANS* (anthocyanin synthase) genes, and three negatively correlated *PPO* (polyphenol oxidase) genes. Overexpression of *CsMYB90* in tobacco resulted in dark-purple transgenic calluses. These results showed that the increased accumulation of three anthocyanins in ZK may promote purple-leaf coloration because of changes in the expression levels of genes, including *CsMYB90*, *F3’Hs*, *ANSs*, and *PPOs*. These findings reveal new insight into the molecular mechanism of anthocyanin biosynthesis in purple-leaf tea plants and provide a series of candidate genes for the breeding of anthocyanin-rich cultivars.

## 1. Introduction

The tea plant (*Camellia sinensis* (L.) O. Kuntze) is one of the most important beverage crops in the world [1]. The color of most tea plant leaves is green, although there are also some special varieties with other leaf colors, such as purple-leaf and white-leaf tea plants [2,3]. The purple-leaf phenotype in tea plants is typically associated with high anthocyanin content; usually, the anthocyanin content is three times higher in purple-leaf tea than that in green-leaf tea [4]. An increasing number of studies have suggested that anthocyanins have antioxidant effects that can significantly reduce the risk of diseases, such as cancer and cardiovascular diseases [5,6]. Therefore, the development of purple-leaf tea varieties has become an important tea breeding target. Recently, several new tea plants with purple leaves have been developed, including Zijuan, Ziyan, and TRFK 306 [7,8,9].

Anthocyanins, a class of water-soluble natural pigments widely found in plants, are one of the main color pigments in plants [10]. More than 600 anthocyanins have been identified in nature, among which cyanidin, delphinidin, pelargonidin, peonidin, petunidin, and malvidin are the six most common anthocyanins in plants [11]. Anthocyanin biosynthesis is a branch of the flavonoid metabolic pathway, and the key enzyme-encoding genes include chalcone synthase (*CHS*), chalcone isomerase (*CHI*), flavanone 3-hydroxylase (*F3H*), flavonoid-3′- hydroxylase (*F3′H*), dihydroflavonol-4-reductase (*DFR*), and anthocyanin synthase (*ANS*) [12]. In recent years, anthocyanin biosynthesis genes, including *CHI*, *CHS*, *F3′H*, and *ANS*, have been identified, and the expression levels of these genes have been found to be strongly associated with anthocyanin accumulation [13,14,15,16,17,18,19].

Transcription factors regulate anthocyanin structural genes that then activate anthocyanin biosynthesis. MYB (v-myb avian myeloblastosis viral oncogene homolog) is considered to be the main determinant of anthocyanin biosynthesis regulation [20,21]. In apple, *MdMYB114* can directly bind to the promoters of *MdANS*, *MdUFGT*, and *MdGST*, thereby promoting the synthesis and transport of anthocyanins [22]. A similar result was reported for eggplant (*Solanum melongena*) *SmMYB113* [23]. In addition, bHLH (basic helix-loop-helix) transcription factors are also important in regulating anthocyanin biosynthesis. In *Dendrobium candidum*, *DcTT8* directly activates the expression of *DcF3′H* and *DcUFGT* by binding to their promoters, thereby regulating anthocyanin biosynthesis [24].

Zikui (*C. sinensis* cv. Zikui) (ZK), a cultivar developed from a purple-bud mutation line of N61 (a line from *C. sinensis* cv. Meitan-Taicha in Guizhou province, China), is a new purple-leaf tea line. The molecular mechanism underlying purple-leaf formation in ZK remains unclear. In this study, we identified the components of the anthocyanin biosynthetic pathways in ZK and N61 with ultra-performance liquid chromatography/electrospray ionization quadrupole (UPLC/ESI-Q) ion trap–tandem mass spectrometry (TRAP-MS/MS). Three anthocyanins were identified, among which petunidin 3-*O*-glucoside was reported for the first time in purple-leaf tea. Then, we investigated key structural and transcription factors controlling purple-leaf characteristics in ZK by integrating metabolomics and transcriptomics analyses. The genes’ expression levels were further examined through quantitative real-time polymerase chain reaction (qRT-PCR). Furthermore, we verified the function of *CsMYB90* through *Agrobacterium*-mediated transformation of tobacco leaf explants. The findings of this study provide new insight into purple-leaf formation in tea plants and reveal a series of candidate genes with applications for the breeding of anthocyanin-rich cultivars.

## 2. Results

### 2.1. Phenotypic Observation and Anthocyanin Content in ZK and N61 in Different Periods

**Figure 1:**
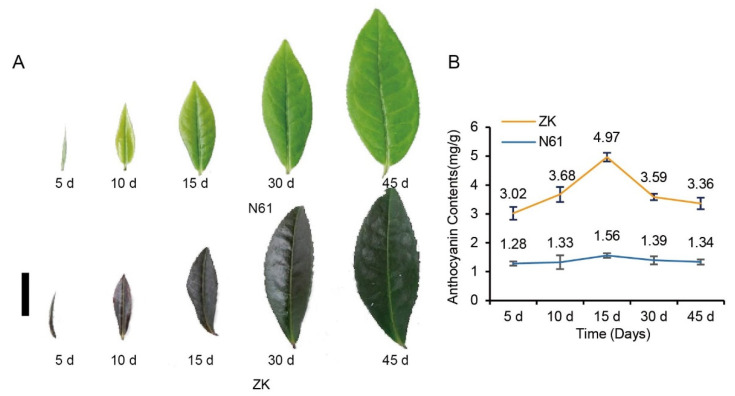
Observation of the purple phenotype and comparison of anthocyanin content changes in Zikui (ZK) and N61 tea cultivars in 5-, 10-, 15-, 30-, and 45-day-old leaves. (**A**) Observation of the purple phenotype, bar = 1 cm. (**B**) Anthocyanin content changes.

In this study, we observed the purple phenotype of the leaves of ZK and N61 in different growth periods. The results showed that the leaves of ZK began to turn purple at 5 days and then gradually became more purple until they were completely dark purple at 15 days. After 15 days, however, the purple faded and became dark green by 45 days. The leaves of N61 remained green throughout the entire duration of the experiment without any obvious change in color. The other characteristics of the leaves of N61 were consistent with those of ZK (Figure 1A).

Furthermore, the total anthocyanins in the leaves of ZK and N61 were measured by the pH differential method. The results showed that the anthocyanin content in the leaves of ZK increased first and then decreased. The anthocyanin content in the leaves of ZK was the highest at 15 days at 4.97 mg/g, which was 3.19 times that of N61 during the same period. It then decreased to 3.59 mg/g at 30 days and 3.36 mg/g at 45 days. In contrast, we did not observe any differences in the anthocyanin content of N61 in the previous five periods, and it was lower than that of ZK in all periods (Figure 1B). These results indicated that anthocyanin content changes were responsible for the color of the tea leaves.

### 2.2. Analysis of Differential Metabolite Components and Relative Contents in ZK and N61

**Figure 2:**
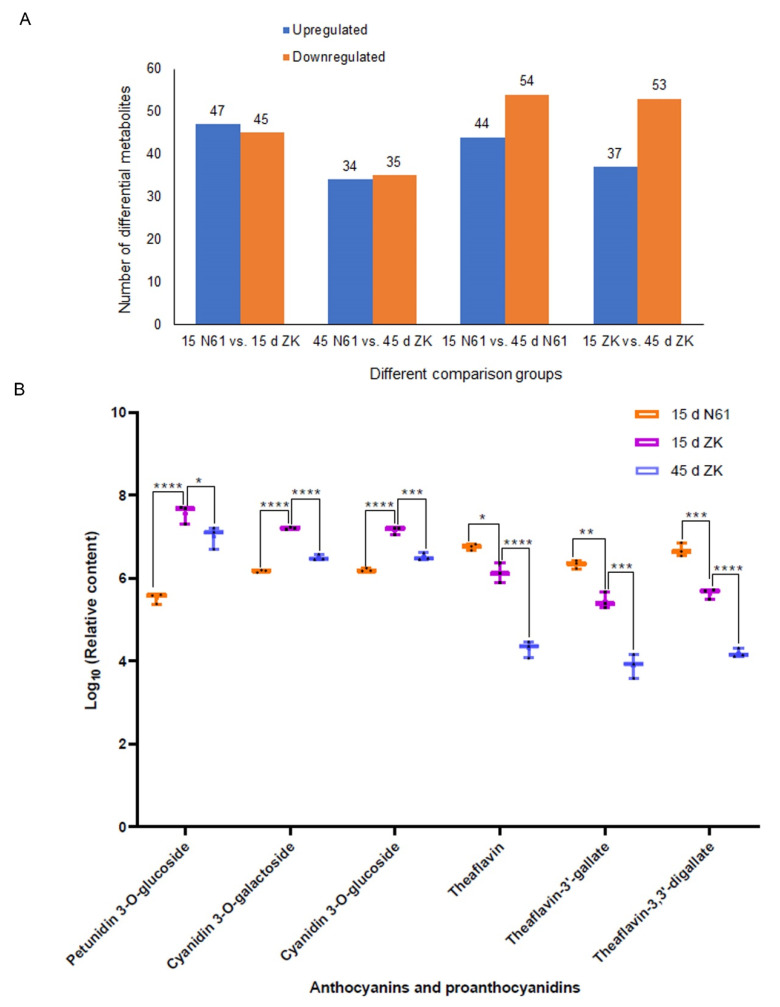
Analysis of differential metabolite components and relative content of key metabolites in the Zikui (ZK) and N61 cultivars. (**A**) Numbers of differential metabolites. (**B**) Relative content of key metabolites in ZK and N61 at 15 and 45 days, presented as dot whisker plots (*n* = 3 independent biological samples; * *p* ≤ 0.05, ** *p* ≤ 0.01, *** *p* ≤ 0.001, **** *p* ≤ 0.0001).

We studied the leaf metabolites of ZK and N61 at 15 and 45 days to detect differentially expressed metabolites (DEMs). A total of 446 metabolites were obtained by liquid chromatography-mass spectrometry (LC-MS) analysis of metabolite components in the leaves of ZK and N61 (Supplementary Dataset S1), including 34 flavonoids, 6 anthocyanins, and 14 proanthocyanidins (Table S1, shown in red). Using the identification criteria of the absolute |log_2_ FC| ≥ 1 and VIP value ≥ 1, 92, 69, 90, and 98 DEMs were identified in the 15-day N61 vs. 15-day ZK, 45-day N61 vs. 45-day ZK, 15-day ZK vs. 45-day ZK, and 15-day N61 vs. 45-day N61, respectively. Among them, 47 and 34 metabolites were upregulated, and 45 and 35 metabolites were downregulated in 15-day N61 vs. 15-day ZK and in 45-day N61 vs. 45-day ZK, respectively (Figure 2A). Kyoto Encyclopedia of Gene and Genome (KEGG) pathway enrichment analysis showed that the DEMs were enriched in many metabolic processes, including the flavonoid and anthocyanin biosynthesis pathways (Q-value < 0.05) (Figure S1).

Quantitative analysis of the differential metabolites showed that petunidin 3-*O*-glucoside was the most abundant among all of the metabolites in the leaves of ZK at 15 days, and its relative content was 3.96 × 10^7^, which was higher than the content of 3.36 × 10^5^ detected in N61 in the same period and the 1.14 × 10^5^ detected in ZK at 45 days. The relative contents of cyanidin 3-*O*-galactoside and cyanidin 3-*O*-glucoside were second only to that of petunidin 3-*O*-glucoside, and their relative contents also followed the same trend (Figure 2B, Tables S2 and S3). This result was consistent with the observation of leaf phenotypes and the relative content of anthocyanins in the leaves of ZK at different stages, indicating that petunidin 3-*O*-glucoside, cyanidin 3-*O*-galactoside, and cyanidin 3-*O*-glucoside were key substances related to the purple-leaf phenotype in ZK.

This study also found that the content of proanthocyanidins, such as the metabolites theaflavin, theaflavin-3’-gallate, and theaflavine-3, 3’-digallate, exhibited the opposite trend to the anthocyanins mentioned earlier (Figure 2B, Tables S2 and S3). More proanthocyanidins were converted to purple anthocyanins at 15 days in the leaves of ZK, resulting in a decrease in the proanthocyanidin content.

### 2.3. Analysis of Transcriptome Sequencing Quality

We performed transcriptome analysis of ZK and N61 using Illumina RNA-seq technology to compare the gene expression profiles of ZK and N61. After the removal of reads containing adaptors and reads with N ratios greater than 10%, all of which were A bases and low-quality reads, more than 49,519,534 clean reads were obtained for both tea plant cultivars. The Q20 percentages (proportion of nucleotides with a quality value larger than 20) and Q30 percentages (proportion of nucleotides with a quality value larger than 30) were above 97.78% and 93.70%, respectively, and the GC content was between 46% and 48% (Table S4).

### 2.4. Analysis of DEGs in ZK and N61

**Figure 3:**
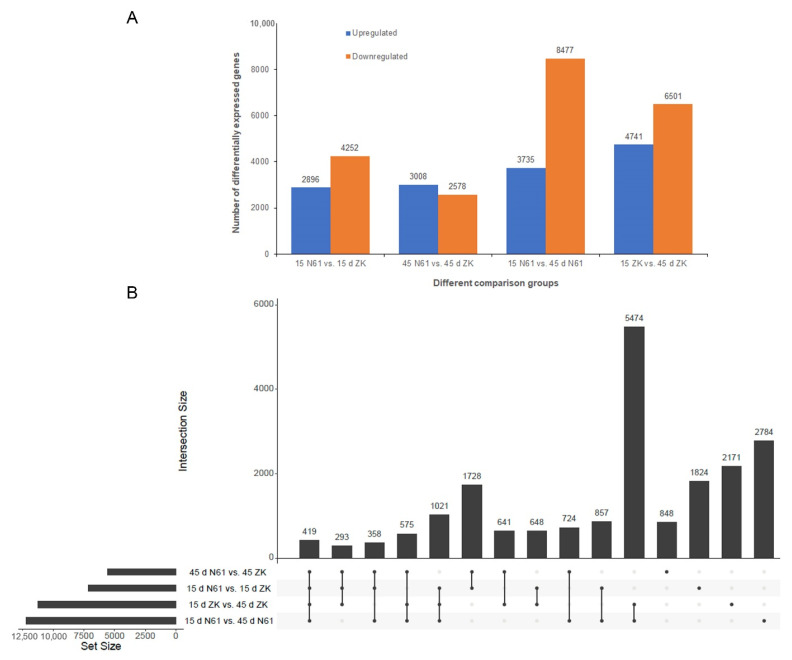
Analysis of differentially expressed genes between the 15- and 45-day stages of the Zikui (ZK) and N61 cultivars. (**A**) Numbers of differentially expressed genes. (**B**) UpSet graphs.

We analyzed the DEGs in the leaves of ZK and N61 at 15 and 45 days. There were 7148, 5586, 12,212, and 11,242 DEGs in the 4 comparison groups (15-day N61 vs. 15-day ZK, 45-day N61 vs. 45-day ZK, 15-day ZK vs. 45-day ZK, and 15-day N61 vs. 45-day N61, respectively). Among them, 2896 and 3008 genes were upregulated, and 4252 and 2578 genes were downregulated in 15-day N61 vs. 15-day ZK and 45-day N61 vs. 45-day ZK, respectively (Figure 3A). The UpSet graphs revealed 419 DEGs that were common to all 4 comparison groups (Figure 3B). The Gene Ontology (GO) analysis assigned 5296, 2653, and 2497 unigenes to the biological processes, cellular components, and molecular function classes, respectively, in the comparison of 15-day N61 vs. 15-day ZK. These genes were further divided into 46 functional subclasses based on their mapped homology (Figure S2A). Then, GO analysis assigned 3252, 1744, and 3464 unigenes to the biological processes, cellular components, and molecular functional classes, respectively, in the comparison of 15-day ZK vs. 45-day ZK (Figure S2B).

KEGG was used to analyze the metabolic pathways related to purple coloring in the leaves of ZK. KEGG analysis revealed flavonoid biosynthesis and phenylpropanoid biosynthesis as the significantly changed pathways in a comparison between 15-day N61 and 15-day ZK. Anthocyanin biosynthesis, flavonoid biosynthesis, and phenylpropanoid biosynthesis were significantly changed compared between 15-day ZK and 45-day ZK (Figure S3).

### 2.5. Candidate Genes for Purple Coloration Obtained by Combined Transcriptome and Metabolome Analysis

**Figure 4:**
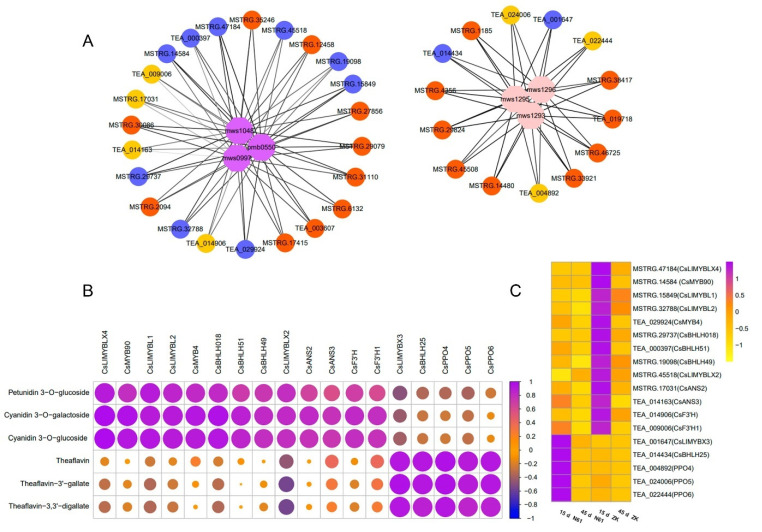
Correlation analysis between genes and key metabolites of purple-leaf formation in Zikui tea. (**A**) Mapping of key metabolites and strongly correlated genes. mws0997, mws1048, and pmb0550 in purple are the metabolites petunidin 3-*O*-glucoside, cyanidin 3-*O*-galactoside, and cyanidin 3-*O*-glucoside, respectively. mws1293, mws1295, and mws1296 in pink are the metabolites theaflavin, theaflavin-3’-gallate, and theaflavine-3, 3’-digallate, respectively. Yellow represents structural genes, and light blue represents transcription factors. Unannotated genes that are highly associated with these metabolites are indicated in orange. The thickness of the line indicates the strength of the correlation. (**B**) Correlation analysis of 18 key genes and key metabolites. (**C**) Heatmap showing the expression levels of 18 key genes, *n* = 3 independent biological samples.

We used the metabolites positively correlated with petunidin 3-*O*-glucoside, cyanidin 3-*O*-galactoside, and cyanidin 3-*O*-glucoside and negatively correlated with the metabolites theaflavin, theaflavin-3’-gallate, and theaflavine-3, 3’-digallate to screen candidate structural genes and transcription factors for purple-leaf formation in ZK using Pearson correlation coefficients (PPC) (Figure 4A).

Among the structural genes, two *F3′H* genes and two *ANS* genes were found to have a strong correlation with petunidin 3-*O*-glucoside, cyanidin 3-*O*-galactoside, and cyanidin 3-*O*-glucoside. The PCCs of *CsF3’H* and *Cs F3’H1* with cyanidin 3-*O*-galactoside and cyanidin 3-*O*-glucoside were 0.83 and 0.80, and 0.78 and 0.81, respectively. The PCCs of *CsANS2* and *CsANS3* with petunidin 3-*O*-glucoside, cyanidin 3-*O*-galactoside, and cyanidin 3-*O*-glucoside were 0.67 and 0.84, 0.79 and 0.60, and 0.78 and 0.75, respectively (Figure 4B, Table S5). In addition, three *PPO* genes were found to be positively correlated (PCC > 0.85) with theaflavin, theaflavin-3’-gallate, and theaflavine-3, 3’-digallate. Among them, the PCCs of *CsPPO4* with theaflavin, theaflavin-3’-gallate, and theaflavin-3, 3’-digallate were 0.90, 0.91, and 0.85, respectively. (Figure 4B, Table S6)

Among the transcription factors, 11 MYB and bHLH transcription factors that were strongly related to key metabolites were identified. Among them, nine *MYB* and *bHLH* transcription factors were strongly correlated with three positively correlated metabolites (PCC > 0.70), in which the correlation coefficients of *CsMYB90* and *CsLIMYBLX4* with petunidin 3-*O*-glucoside, cyanidin 3-*O*-galactoside, and cyanidin 3-*O*-glucoside were 0.80 and 0.96, 0.92 and 0.90, and 0.99 and 0.98, respectively, and the PCCs of *CsBHLH51* with petunidin 3-*O*-glucoside, cyanidin 3-*O*-galactoside, and cyanidin 3-*O*-glucoside were 0.71, 0.87, and 0.86, respectively. Another two MYB and BHLH transcription factors were strongly associated with three negatively correlated metabolites (PPC > 0.87). The PCCs of *CsLIMYBX3* and *CsBHLH25* with theaflavin, theaflavin-3’-gallate, and theaflavine-3, 3’-digallate were 0.93 and 0.96, 0.94 and 0.92, and 0.93 and 0.88, respectively (Figure 4B, Tables S5 and S6).

The expression levels of genes strongly positively correlated with metabolites in ZK at 15 days were higher than those in N61 and were also higher than those in ZK at 45 days. Conversely, the expression levels of genes strongly negatively correlated with metabolites in N61 were higher than those in ZK in the same period (Figure 4C, Tables S7 and S8). The expression levels of these genes were consistent with the observed change trend in leaf phenotype, suggesting that their expression was closely related to purple-leaf formation in ZK. In addition, we found that many unannotated genes also had a strong correlation with positively and negatively correlated metabolites (Tables S9 and S10), and the changing trend in the expression level at each stage was consistent with the changing trend in anthocyanin content (Figure 1, Tables S7 and S8). These genes may also be related to purple-leaf formation in ZK. Their functions remain to be investigated.

### 2.6. Regulation of Anthocyanidin Biosynthesis and Accumulation

**Figure 5:**
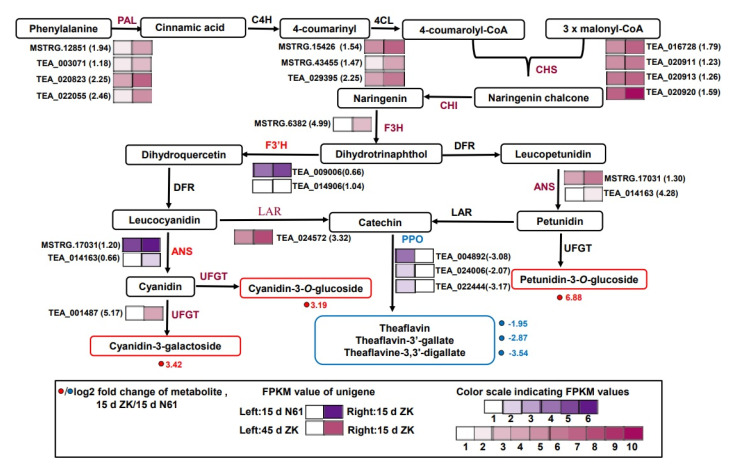
Transcriptional profiling of genes involved in the phenylpropane and flavonoid biosynthesis pathways in Zikui (ZK) and N61 tea cultivars. Grids with a purple color scale from light to dark represent fragments per kilobase of exon per million fragments mapped (FPKM) values of 0–10, 10–20, 20–40, 40–80, 80–160, and 160–320. Grids with a red color scale from light to dark represent FPKM values of 0–10, 10–20, 20–40, 40–80, 80–160, 160–320, 320–640, 640–1280, 1280–2560, and more than 2560. *PAL*, phenylalanine ammonia-lyase; *C4H*, cinnamic acid 4-hydroxylase; *4CL*, 4-coumarate CoA ligase; *CHS*, chalcone synthase; *CHI*, chalcone isomerase; *F3H*, flavanone 3-hydroxylase; *F3′H*, flavonoid 3′-hydroxylase; *DFR*, dihydroflavonol 4-reductase; *ANS*, anthocyanidin synthase; *UFGT*, *UDP* glucose-flavonoid 3-*O*-glcosyl-transferase; *LAR*, leucocyanidin reductase.

At 15 days, the *F3’H* and *ANS* genes exhibited upregulated expression in ZK compared with N61. *CsF3’H* and *CsF3’H1* were upregulated by 1.04- and 0.66-fold, respectively, and *CsANS2* and *CsANS3* were upregulated by 1.20- and 0.77-fold, respectively. This was consistent with the result that the contents of three metabolites, namely petunidin 3-*O*-glucoside, cyanidin 3-*O*-galactoside, and cyanidin 3-*O*-glucoside, were higher than those of N61 in ZK (Figure 5). One of the causes of anthocyanin degradation is polyphenol oxidase (PPO), which can also oxidize catechins to produce theaflavin. In this study, we found that the three *PPO* genes, *CsPPO4*, *CsPPO5*, and *CsPPO6,* were downregulated by −3.08-, −2.07-, and −3.17-fold, respectively. This was consistent with the decrease of theaflavin, theaflavin-3’-gallate, and theaflavine-3, 3’-digallate in ZK (Figure 5).

High upregulation and high fragments per kilobase of exon per million fragments mapped (FPKM) values for the genes indicated the enhanced biosynthesis of flavonoids and anthocyanidins. Most of the structural genes were upregulated in ZK at 15 days compared with at 45 days. Four *PAL* genes were upregulated by 1.94-, 1.18-, 2.25-, and 2.46-fold. Four *CHS* genes were upregulated by 1.79-, 1.23-, 1.26-, and 1.59-fold. Three *CHI* genes were upregulated by 1.54-, 1.47-, and 2.26-fold, and one *F3H* gene was upregulated 4.98-fold. Two *ANS* genes were upregulated by 1.29- and 4.28-fold, and one *UFGT* gene was upregulated by 5.17-fold (Figure 5, Table S11). The upregulated expression of these genes, especially the two *ANS* genes and UFGT genes, could largely explain the high accumulation of petunidin 3-*O-*glucoside, cyanidin 3-*O*-galactoside, and cyanidin 3-*O*-glucoside at 15 days.

### 2.7. Quantitative RT-PCR Validation of the Transcriptomic Data

**Figure 6:**
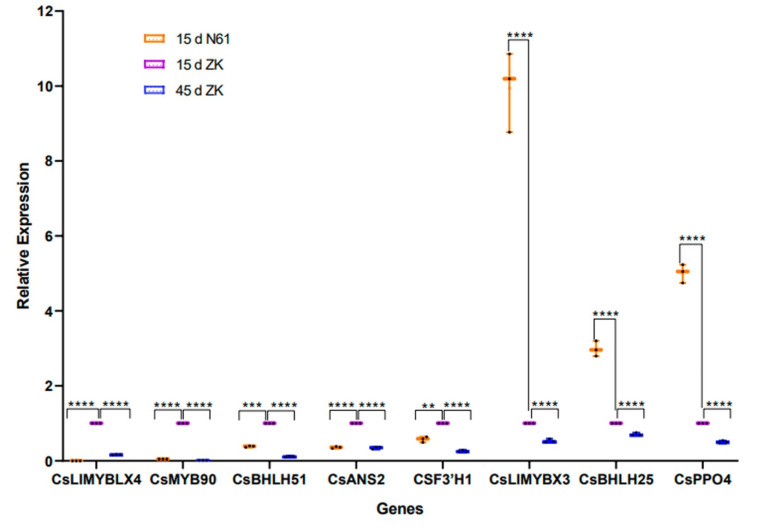
Expression of representative genes in Zikui (ZK) and N61 tea cultivars validated by quantitative real-time polymerase chain reaction (qRT-PCR). Presented as dot and whisker plots (*n* = 3 independent biological samples; ** *p* ≤ 0.01, *** *p* ≤ 0.001, **** *p* ≤ 0.0001).

To verify the credibility of the transcriptome information, eight genes related to anthocyanin metabolism were selected for validation by qRT-PCR. The qRT-PCR results showed that the expression patterns of these genes were very similar to the RNA-seq results (Figure 6).

### 2.8. Functional Analysis of the CsMYB90 Gene in Nicotiana Tabacum

**Figure 7:**
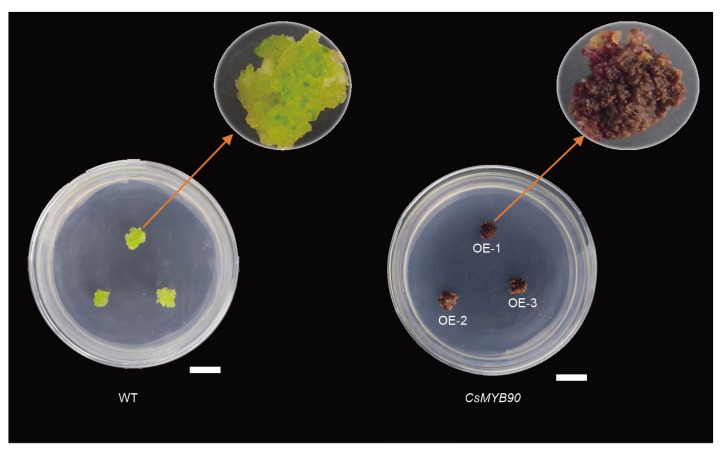
Overexpression of *CsMYB90* resulted in visible dark-purple coloration in the calluses of transgenic tobacco. Bar = 1 cm.

PCAMBIA-super1300-35S:CsMYB90 was introduced into tobacco K326 (*N. tabacum* K326). The expression level of *CsMYB90* in calluses was measured through qRT-PCR. The results showed that the *CsMYB90* gene was detected in purple calluses but not in white calluses (Figure 7, Figure S4). This indicated that overexpression of the *CsMYB90* gene in tobacco could promote anthocyanin synthesis and form purple calluses.

## 3. Discussion

High anthocyanin contents cause the purple-leaf phenotype in tea plants. Generally, the content of anthocyanins in tea leaves accounts for about 0.01% of the dry matter weight, whereas the content of anthocyanins in purple bud tea can reach up to 0.5–1.0% [25]. A previous study found that the content of anthocyanins in Ziyan tea plants was 2.29 mg/g using the pH differential method [26]. In this study, the highest anthocyanin content determined by the pH differential method was 4.97 mg/g at 15 days, which was higher than that determined by Tian et al. This result indicated that ZK is a typical purple-leaf tea. In addition, the anthocyanin content in the leaves of ZK increased first and then decreased in each period, being higher than that of N61 in the same period. This finding was consistent with the observation of the purple phenotype in the leaves. Therefore, we used the leaves at 15 days, which exhibited the deepest purple color and had the highest anthocyanin contents in ZK, for anthocyanin metabolism and transcriptome analysis.

Anthocyanins in their free state are rare under natural conditions, and usually form anthocyanins with glucose and galactose through glycosidic bonds [27]. Studies have found that the anthocyanins in Zijuan are mainly cyanidin-3-*O*-galactoside, delphinium-3-*O*-(6-coumaroyl)-galactoside, and cyanidin-3-*O*-(6-coumaroyl)-galactoside, which are primarily concentrated in the bud leaves, and the content of anthocyanins in the mature leaves is lower [28]. The anthocyanin components in the bud leaves of Jinmingzao purple-leaf tea are mainly peonidin 3-*O*-glucoside chloride, cyanidin 3-*O*-glucoside, and delphinidin 3-*O-*glucoside, and are higher than those in green-leaf tea, at 13.39, 6.78, and 6.73 times higher, respectively [29]. In this study, six anthocyanin components were detected in ZK, including petunidin 3-*O*-glucoside, cyanidin 3-*O*-galactoside, cyanidin 3-*O*-glucoside, jaceosidin, cyanin chloride, and cyanidin 3-rutinoside. During the critical period (15 days) of purple-leaf formation in ZK, the petunidin 3-*O*-glucoside content was the highest (3.96 × 10^7^). This is the first time that this anthocyanin has been detected in purple-leaf tea. Cyanidin 3-O-galactoside content was the next highest (1.58 × 10^7^), followed by cyanidin 3-*O*-glucoside (1.44 × 10^7^). The contents of petunidin 3-*O*-glucoside, cyanidin 3-*O*-galactoside, and cyanidin 3-*O*-glucoside were higher than those in N61, at 117.76, 10.68, and 9.138 times those of N61 in the same period, respectively. We speculated that petunidin 3-*O*-glucoside, cyanidin 3-*O*-galactoside, and cyanidin 3-*O*-glucoside were the key metabolic substances for purple-leaf formation in ZK.

Theaflavins are compounds with a benzophenone structure. They are generated through the oxidative condensation of catechins and their derivatives [30]. In the present study, we found that the content of theaflavins was negatively correlated with anthocyanins at 15 days. We speculated that catechin, the precursor of theaflavins, shared a common synthetic pathway with anthocyanins. Under the same conditions, higher anthocyanin contents in ZK resulted in lower catechin, which led to the theaflavin contents in ZK being lower than those in N61.

Many structural genes are involved in the biosynthesis of anthocyanins, including the early structural genes *PAL*, *C4H*, *4CL*, *CHS*, *CHI*, *F3H*, and *F3’H*, and the late structural genes *DFR*, *LAR*, and *ANS*. A previous study has shown that most of these structural genes are highly expressed in the tissues of purple plants [31]. In the present study, most structural genes involved in anthocyanin biosynthesis during purple-leaf formation in ZK were upregulated in the new leaves (15 days) compared with the old leaves (45 days), which was similar to the findings of a previous study in the Zijuan tea cultivar [32,33]. When comparing ZK with N61 at 15 and 45 days, the early structural gene *F3’H* and the late structural gene *ANS* showed differences and strong correlations with petunidin 3-*O*-glucoside, cyanidin 3-*O*-galactoside, and cyanidin 3-*O*-glucoside (PPC > 0.6). We did not observe, however, any difference in the expression levels of other early or late biosynthetic genes. *F3’H* determines the type of anthocyanin by determining the type of B-ring hydroxylation and is a key enzyme in the anthocyanin synthesis pathway. A high expression level of *F3’H* will provide a sufficient amount of the precursor dihydroquercetin for the biosynthesis of flavonols and anthocyanins [16]. It has been found that overexpression of the *HbF3’H1* gene in tobacco can increase anthocyanin contents in the petals of transgenic tobacco and significantly deepen their color [34]. In addition, *ANS* is an enzyme that catalyzes the synthesis of colored anthocyanins from leucoanthocyanidin. One study found that the expression level of *ANS* in the purple-red leaves of *Paeonia* × *suffruticosa* was higher than that in the yellow-green leaves [35]. Other studies have shown that the overexpression of *SmANS* increased the anthocyanin content in *Salvia miltiorrhiza*, whereas the low expression of the *ANS* gene in the white flowers of *S. miltiorrhiza* was attributed to the white coloring [18,19]. The upregulated expression of *F3’H* and *ANS* might promote the accumulation of anthocyanins in ZK tea plants.

PPO is considered to be one of the causes of anthocyanin degradation. Studies have shown that the PPO active enzyme is the main factor leading to anthocyanin degradation in juice prepared from red muscadine grapes [36]. In the present study, three PPO enzyme genes were downregulated during the purple stage (15 days) of ZK leaves and showed a stronger negative correlation with three key metabolites, including petunidin 3-*O-*glucoside, cyanidin 3-*O-*galactoside, and cyanidin 3-*O*-glucoside. This result suggested that the enzymes encoded by the *PPO* gene could degrade anthocyanins in ZK.

Transcription factors such as MYB and bHLH play an important role in regulating anthocyanin formation. In the tea plant, *CsMYB6A* can significantly upregulate *CHS* and *A3T*, thus promoting the accumulation of flavonoids in purple buds and leaves [37]. Another study indicated that the transcription factor R2R3-MYB anthocyanin 1 (*CsAN1*) induced the specific accumulation of anthocyanins in the young leaves and stems of Zijuan [38]. In peony, *PsbHLH* was involved in the regulation of anthocyanin biosynthesis and accumulation by directly activating the promoters of *PsANS* and *PsDFR* [39]. Herein, seven MYB and four bHLH transcription factors were discovered. Among them, six were upregulated and positively correlated with three key metabolites. *CsMYB90* was strongly correlated with petunidin 3-*O*-glucoside, cyanidin 3-*O*-galactoside, and cyanidin 3-*O*-glucoside, with PPC values of 0.80, 0.96, and 0.92, respectively. A gene designated *CsLIMYBX3* was negatively correlated with anthocyanin accumulation, and its correlation coefficients with the negatively correlated metabolites theaflavin, theaflavin-3’-gallate, and theaflavin-3, 3’-digallate were 0.93, 0.96, and 0.94, respectively. Among the *bHLH* transcription factors, three were upregulated and positively correlated with three key metabolites, whereas one bHLH was negatively correlated with anthocyanin accumulation. In addition, the overexpression of the *CsMYB90* gene in tobacco resulted in the transgenic tobacco calluses presenting a dark-purple color compared with the wild-type calluses. These results indicated that the changes in expression of these transcription factor genes could be a factor in purple-leaf formation in ZK.

## 4. Materials and Methods

### 4.1. Plant Materials

The ZK and N61 tea plants were grown in the natural environment of the tea plant germplasm resource nursery of the Institute of Agricultural Biotechnology, South Campus of Guizhou University, Huaxi District, Guiyang City, Guizhou Province, China (E106°67′ N:26°33′; altitude: 1055–1100 m). The tobacco used for transformation was grown on MS medium in the tissue culture room (temperature 25 °C; air humidity 80%; light intensity 3000 lx; light/dark: 14 h/10 h) of the Institute of Agricultural Biotechnology, South Campus of Guizhou University. Under natural conditions, the leaves of ZK and N61 at 5, 10, 15, 30, and 45 days of growth were collected for phenotypic observation and total anthocyanin determination. The 15- and 45-day samples were frozen in 10 mL of liquid nitrogen and stored at −80 °C for RNA and metabolite extraction. All of the experiments were performed with three biological replicates.

### 4.2. Determination of Anthocyanin Content in the Leaves at Different Stages

The leaves (0.3 g) of ZK and N61 at the five periods were ground in liquid nitrogen and then immersed in 5 mL of 1% HCl/ethanol solution for 24 h, at 4 °C. The tissue homogenates were oscillated for 30 s and centrifuged at 4 °C and 4000 rpm for 10 min, and the absorbance of the supernatants was measured at a wavelength of 510 nm using an ultraviolet spectrophotometer (UV-8000, METASH). The relative anthocyanin concentration was calculated using the following formula: anthocyanin content (mg/g) = (A510/Εl) × MW × DF × V/Wt, where L represents the optical path (1 cm), DF represents the solution dilution factor, V represents the total volume of diluted solution (mL), Wt represents the sample quality (g), MW represents the molecular weight of delphinidin glucoside (465.2), and ε represents the extinction coefficient of delphinidin glucoside (29,000). The content of delphinidin glucoside (Del-3-glc) was determined by using it as the standard substance.

### 4.3. Metabolite Extraction

The freeze-dried leaves were crushed using a mixer mill (MM 400, Retsch, Shanghai, China) with a zirconia bead for 1.5 min at 30 Hz. One hundred milligrams of powder were weighed and extracted overnight at 4 °C in 1.0 mL of 70% aqueous methanol. Following centrifugation at 10,000× *g* for 10 min, the extracts were absorbed (CNWBOND Carbon-GCB SPE Cartridge, 250 mg, 3 mL; ANPEL, Shanghai, China, www.anpel.com.cn/cnw (accessed on 29 August 2019)) and filtrated (SCAA-104, 0.22 μm pore size; ANPEL, Shanghai, China, http://www.anpel.com.cn/ (accessed on 29 August 2019)) before LC-MS analysis.

### 4.4. High-Performance Liquid Chromatography Conditions

We used UPLC (Shim-pack UFLC SHIMADZU CBM30A, http://www.shimadzu.com.cn/ (accessed on 29 August 2019)) and MS/MS (Applied Biosystems 6500 QTRAP, Waltham, MA, USA, http://www.appliedbiosystems.com.cn/ (accessed on 29 August 2019)) to analyze the sample extracts. The UPLC analysis was performed under the following conditions. UPLC: column, Waters (Shanghai, China) ACQUITY UPLC HSS T3 C18 (1.8 μm, 2.1 mm × 100 mm); solvent system, water (0.04% acetic acid): acetonitrile (0.04% acetic acid); gradient elution program, 95:5 V/V at 0 min, 5:95 V/V at 11.0 min, 5:95 V/V at 12.0 min, 95:5 V/V at 12.1 min and 95:5 V/V at 15.0 min; flow rate, 0.40 mL/min; column temperature, 40 °C, and an injection volume of 2 μL. The separated samples were connected to electrospray ionization (ESI)-QTRAP-MS for mass spectrometric analysis.

### 4.5. ESI-QTRAP-MS/MS

Linear ion hydrazine-flight time (LIT) and triple quadrupole (QQQ) scans were acquired on a triple quadrupole-linear ion trap mass spectrometer (API 6500 Q TRAP LC/MS/MS System) equipped with an ESI Turbo Ion-Spray interface, operating in positive ion mode and controlled by Analyst 1.6.3 software (AB Sciex, Shanghai, China). The ESI source operation parameters were as follows: Ion source, turbo spray; source temperature, 500 °C; ion spray voltage (IS), 5500 V. The ion source gas I (GSI), gas II (GSII), and curtain gas (CUR) were set at 55, 60, and 25.0 psi, respectively, and the collision gas (CAD) was high. Instrument tuning and mass calibration were performed with 10 and 100 μmol/L polypropylene glycol solutions in QQQ and LIT modes, respectively. The QQQ scans were acquired as multiple reaction monitoring (MRM) experiments with the collision gas (nitrogen) set to 5 psi. De-clustering potential (DP) collision energy (CE) measurements for individual MRM transitions were performed with further DP and CE optimization. A specific set of MRM transitions was monitored for each period according to the metabolites eluted within this period.

### 4.6. Identification and Quantitative Analysis of Metabolites

We performed data filtering, peak detection, alignment, and calculations using Analyst 1.6.1 software. To produce a matrix containing less biased and redundant data, peaks were checked manually for signal/noise (*s/n*) > 10, and in-house software written in Perl was used to remove the redundant signals caused by different isotopes, in-source fragmentation, K^+^, Na^+^, and NH4^+^ adducts, and dimerization. To facilitate the identification/annotation of metabolites, accurate m/z for each Q1 was obtained. Total ion chromatograms (TICs) and extracted ion chromatograms (EICs or XICs) of QC samples were exported to provide an overview of metabolite profiles of all samples. The area of each chromatographic peak was calculated. Peaks were aligned across the different samples based on the spectral pattern and retention time. Metabolites were identified by searching the self-built MWDB database (Metware Biotechnology Co., Ltd., Wuhan, China) and public databases (MassBank, KNApSAcK, HMDB [40] MoTo DB, and METLIN [41]) and comparing the *m/z* values, retention time, and fragmentation patterns with the standards. Unsupervised principal component analysis (PCA) (Figure S5A), hierarchical cluster analysis (HCA) (Figure S5B), and partial least-squares discriminant analysis (OPLS-DA) were performed using the statistics function prcomp within R (www.r-project.org (accessed on 29 August 2019)). We applied the variable importance in projection (VIP) score of the OPLS model to rank the metabolites that best distinguished the two groups [42]. The threshold of VIP was set to 1. In addition, t-tests were also used as a univariate tool to screen differential metabolites. Significantly different metabolites between groups were determined based on the *p*-value of the *t*-test < 0.05, VIP ≥ 1, and an absolute Log_2_FC (fold change) ≥ 1.

### 4.7. RNA Extraction and Illumina Sequencing

Total RNA was extracted using a TRIzol reagent kit (Invitrogen, Carlsbad, CA, USA) according to the manufacturer’s protocol. The RNA quality was assessed on an Agilent 2100 Bioanalyzer (Agilent Technologies, Palo Alto, CA, USA) and checked using RNase-free agarose gel electrophoresis. After the total RNA was extracted, eukaryotic mRNA was enriched by Oligo (Dt) beads. Then, the enriched mRNA was fragmented into short fragments using fragmentation buffer and reverse-transcribed into cDNA using an NEBNext Ultra RNA Library Prep Kit for Illumina (NEB #7530, New England Biolabs, Ipswich, MA, USA). The purified double-stranded cDNA fragments were end-repaired, subjected to A base addition, and ligated to Illumina sequencing adapters. The ligation reaction was purified with AMPure XP Beads (1.0X). Ligated fragments were subjected to size selection by agarose gel electrophoresis and PCR amplification. The resulting cDNA library was sequenced using an Illumina NovaSeq6000 by Gene Denovo Biotechnology Co. (Guangzhou, China).

### 4.8. RNA Sequencing Data Analysis and Annotation

To acquire high-quality clean reads, the raw reads were further filtered by fastp (version 0.18.0) [43]. We obtained clean reads from the raw data by removing reads containing adapters, reads containing more than 10% unknown nucleotides (N), and low-quality reads containing more than 50% low-quality (Q-value ≤ 20) bases. All downstream analyses were based on clean, high-quality data. Gene functions were annotated using the KEGG pathway database, the National Center for Biotechnology Information (NCBI) non-redundant (Nr) database, the Swiss-Prot protein database, the Eukaryotic Clusters of Orthologous Groups (KOG) database, the GO database, the Pfam database, and the NCBI nucleotide sequences (Nt) database.

The levels of gene expression were estimated by RSEM [44]. Analysis of the DEGs of the two groups was performed with DESeq2 [45], which included standardizing the read-count, calculating a hypothesis testing probability (*p*-value), and conducting multiple hypothesis testing corrections to obtain the false discovery rate (FDR) value (Hochberg FDR). The results of all statistical tests were corrected by multiple tests using the hypothesis test *p*-value and Hochberg FDR. Genes were determined to be significantly differentially expressed at FDR < 0.05 and |log_2_ FC| ≥ 1 according to DESeq. The GO enrichment analysis of the DEGs was implemented according to the GO database (http://www.geneontology.org/ (accessed on 19 August 2019)). Pathway analysis elucidated significant pathways of DEGs according to the KEGG database (http://www.genome.jp/kegg/ (accessed on 19 August 2019)).

### 4.9. Integrated Metabolome and Transcriptome Analysis

For the combined analysis of metabolome and transcriptome data, the COR program from R was used to calculate the value of the PCC in this study. The corresponding correlation network analysis was visualized with the Cytoscape software (version 3.7.0).

### 4.10. Quantitative RT-PCR

A total of eight genes were differentially expressed and subjected to qRT-PCR verification. Total RNA was extracted using the Q341 RT-PCR reagent (Vazyme Biotech, Nanjing, China). The cDNA was synthesized according to the instructions of the Reverse Transcription Kit (R223) (Vazyme Biotech, Nanjing, China). The *TUBA3* gene was used as an internal reference. The primer pairs were designed using Primer Premier 5.0 software. The specific primers for genes involved in anthocyanin biosynthesis and the *TUBA3* gene are listed in Table S12. Each biological sample was tested in triplicate, and the standard deviation (SD) values of the means were calculated using standard statistical methods. The expression of genes was analyzed using the ΔΔCt data analysis method, and gene relative expression was calculated using the 2^-ΔΔCt^ method.

### 4.11. Transformation of Tobacco Plants with CsMYB90

Two specific primers (Table S12) were designed using the Primer Premier 5.0 software and synthesized by Biochemical Bioengineering Co., Ltd. (Shanghai, China). The PCAMBIA-super1300 plasmid was subjected to double digestion with *XbaI* and *PstI*, following which the PCR products and double digestion products were recovered. The target gene was ligated with the PCAMBIA-super1300 overexpression vector. The PCAMBIA-super1300-35S:CsMYB90 fusion expression vector was transformed into *Escherichia coli* DH5α competent cells, and then the positive clones were identified by PCR. After the plasmid containing the target gene was extracted, it was transformed into *Agrobacterium* GV3101 competent cells. The detection primers were the same as the amplification primers. The PCR conditions used were as follows: pre-denaturation (94 °C, 2 min), 35 cycles of denaturation (94 °C, 30 s), annealing (55 °C, 30 s), extension (72 °C, 1 min), and a final extension (72 °C, 10 min). The positive monoclonal colonies were preserved. A single colony containing each target construct was confirmed by PCR and then used for the genetic transformation of tobacco. The *Agrobacterium*-mediated method was used for tobacco transformation with 5 mg/L of hygromycin selection. *Agrobacterium* was cultured with 50 mL of YEP medium (without agar, pH = 7.2) containing 100 mg/L of kanamycin and 50 mg/L of rifampicin at 28 °C until an optical density at 600 nm (OD_600_) of 0.8 was achieved. Precultured leaves were trimmed into 1 cm × 1 cm pieces using scalpels. The prepared *Agrobacterium* inoculums were separately transferred to sterile Petri dishes and trimmed leaf explants were inoculated for 8 min. The *Agrobacterium*-treated explants and leaves were blotted on sterile filter paper and placed with the adaxial side up onto cocultivation agar medium (Murashige–Skoog (MS) medium supplemented with 1 mg/L of 6-benzylaminopurine (BA), 0.1 mg/L of α-naphylacetic acid (NAA), 8 g/L of agar, and 100 mg/L of acetosyringone (AS), pH = 5.2), overlaid with a single piece of sterile filter paper for 48 h in the dark at 28 °C. After the co-cultivation period, the explants were transferred to selection agar medium (MS supplemented with 1 mg/L of 6-BA, 0.1 mg/L of NAA, 100 mg/L of timentin, and 5 mg/L of hygromycin, pH = 5.8). The selection agar medium was changed once a week. Transgenic calluses were obtained after four weeks of growth. We measured the expression level of *CsMYB90* in transgenic calluses (OE-1, OE-2, and OE-3) through qRT-PCR.

### 4.12. Statistical Analysis

Each experiment was set up with three biological repeats, and all of the data were expressed as the mean ± SD. One-way analysis of variance and t-tests were performed with GraphPad Prism8.0.lnk. Significant differences between groups are indicated by asterisks (*). The number of asterisks (*) represents the degree of difference.

## 5. Conclusions

In this study, we explored the molecular mechanism underlying the purple-leaf characteristic in ZK by integrating metabolomic and transcriptomic analyses. The results showed that petunidin 3-*O*-glucoside, cyanidin 3-*O*-galactoside, and cyanidin 3-*O-*glucoside were the major anthocyanins related to the purple-leaf phenotype in ZK. Eleven transcription factors were identified, among which *CsMYB90* had a strong correlation with the three key anthocyanins (PPC > 0.8). Furthermore, we also identified key correlated structural genes, including two positively correlated *F3’H* genes, two positively correlated *ANS* genes, and three negatively correlated *PPO* genes. The overexpression of *CsMYB90* in tobacco resulted in a dark-purple color in transgenic calluses. These results not only provided new insight into the molecular mechanism of anthocyanin biosynthesis in purple-leaf tea plants, but also provided a series of candidate genes for the molecular breeding of tea plants with increased anthocyanin content.

## Data Availability

All data generated or analyzed during this study are included in this published article and its Supplementary Materials. The raw RNA-seq data reported in this article have been deposited in the NCBI SRA (accession numbers: SRP370607).

## Supplementary Materials

The following supporting information can be downloaded at https://www.mdpi.com/article/10.3390/ijms23094780/s1.
