# Supplementary material for: Integrative Analysis of Metabolomics and Transcriptomics Reveals Molecular Mechanisms of Anthocyanin Metabolism in the Zikui Tea Plant (Camellia sinensis cv. Zikui)
Source: Int J Mol Sci. 2022 Apr 26;23(9):4780. doi: 10.3390/ijms23094780 (PMC9103729; doi:10.3390/ijms23094780)

Supplementary Table S1. Classification and identification of differential metabolites in Zikui (ZK) and N61 tea plants.

Type	Number	Percentage
All	446	100
Phenolic acids	73	16.368
Amino Acid And Its Derivatives	54	12.108
**Flavonoid**	**34**	**7.623**
Nucleotide and its derivates	30	6.726
Organic acids	29	6.502
Flavonols	27	6.054
Alcohols and polyols	23	5.157
Free fatty acids	23	5.157
Flavonoid carbonoside	19	4.26
flavanols	15	3.363
LPC	14	3.139
Others	14	3.139
**Proanthocyanidins**	**14**	**3.139**
Alkaloids	11	2.466
Lignans	8	1.794
Dihydro flavonoid	7	1.57

Glycerol ester	7	1.57
**Anthocyanins**	**6**	**1.345**
Dihydroflavonol	5	1.121
LPE	5	1.121
Isoflavones	4	0.897
Phenolamine	4	0.897
vitamin	4	0.897
Coumarins	3	0.673
Sphingolipids	3	0.673
Chalcone	2	0.448
PC	2	0.448
Plumerane	2	0.448
Orange Flavonoids	1	0.224
Steroid	1	0.224
Tannin	1	0.224
Xanthone	1	0.224

Supplementary Table S2. Anthocyanin and proanthocyanidin metabolites content in Zikui (ZK) and N61 tea plants in 15-d-old leaves.

Index	15 d N61	15 d ZK	log2_FC(15 d ZK / 15 d N61)	Compounds	Class II
mws0997	3.36E+05	3.96E+07	6.88	Petunidin 3-*O*-glucoside	Anthocyanins
mws1048	1.48E+06	1.58E+07	3.42	Cyanidin3-*O*-galactoside	Anthocyanins
pmb0550	1.58E+06	1.44E+07	3.19	Cyanidin 3-*O*-glucoside	Anthocyanins
pmp000004	6.38E+05	2.83E+06	2.15	Jaceosidin	Anthocyanins
mws0171	6.37E+06	8.18E+06	0.36	Cyanin chloride	Anthocyanins
mws0169	3.41E+06	3.18E+06	-0.10	Cyanidin 3–rutinoside	Anthocyanins
mws0836	8.54E+05	7.51E+05	-0.18	Procyanidin B1	Proanthocyanidins
pmb0837	1.33E+05	8.67E+04	-0.62	Procyanidin A3	Proanthocyanidins
pmn001647	6.80E+05	4.07E+05	-0.74	procyanidin C2	Proanthocyanidins
pmb2947	1.45E+06	8.18E+05	-0.82	Catechin-catechin-catechin	Proanthocyanidins
pmp000280	2.17E+06	1.20E+06	-0.86	procyanidin C-2	Proanthocyanidins
pme0436	3.26E+07	1.71E+07	-0.93	Procyanidin B3	Proanthocyanidins
pmp000275	5.99E+04	3.14E+04	-0.93	gambiriin A-1	Proanthocyanidins
pmp000274	1.21E+07	5.59E+06	-1.11	procyanidin B-3	Proanthocyanidins
pmn001646	5.15E+05	1.83E+05	-1.49	procyanidin C1	Proanthocyanidins
mws1293	5.71E+06	1.47E+06	-1.95	Theaflavin	Proanthocyanidins

pmp000273	1.55E+05	2.57E+04	-2.59	gambiriin B-3	Proanthocyanidins
mws1295	2.19E+06	2.99E+05	-2.87	Theaflavin-3'-gallate	Proanthocyanidins
mws1294	2.82E+06	3.79E+05	-2.89	Theaflavin-3-gallate	Proanthocyanidins
mws1296	5.02E+06	4.31E+05	-3.54	Theaflavin-3-3'-digallate	Proanthocyanidins

Key metabolites are enclosed in red boxes.

Supplementary Table S3. Anthocyanin and proanthocyanidin metabolite content in Zikui (ZK) tea plant at 15 d and 45 d.

Index	15 d *ZK*	45 d ZK	log2_FC(15 d ZK /45 d ZK)	Compounds	Class II
mws1293	1.47E+06	2.12E+04	6.12	Theaflavin	Proanthocyanidins
mws1294	3.79E+05	7.66E+03	5.63	Theaflavin-3-gallate	Proanthocyanidins
mws1295	2.99E+05	8.90E+03	5.07	Theaflavin-3'-gallate	Proanthocyanidins
mws1296	4.31E+05	1.58E+04	4.77	Theaflavin-3-3'-digallate	Proanthocyanidins
mws1048	1.58E+07	3.20E+06	2.30	Cyanidin 3-*O*-galactoside	Anthocyanins
pmb0550	1.44E+07	3.32E+06	2.12	Cyanidin 3-*O*-glucoside	Anthocyanins
mws0997	3.96E+07	1.14E+07	1.80	Petunidin 3-*O*-glucoside	Anthocyanins
pmp000004	2.83E+06	1.28E+06	1.14	Jaceosidin	Anthocyanins
pmb2947	8.18E+05	5.89E+05	0.47	Catechin-catechin-catechin	Proanthocyanidins
pmb0837	8.67E+04	6.67E+04	0.38	Procyanidin A3	Proanthocyanidins
mws0836	7.51E+05	7.34E+05	0.03	Procyanidin B1	Proanthocyanidins
pmp000280	1.20E+06	1.18E+06	0.02	procyanidin C-2	Proanthocyanidins
pmp000274	5.59E+06	5.69E+06	-0.03	procyanidin B-3	Proanthocyanidins
pmn001646	1.83E+05	2.35E+05	-0.36	procyanidin C1	Proanthocyanidins
pme0436	1.71E+07	2.46E+07	-0.52	Procyanidin B3	Proanthocyanidins
pmn001647	4.07E+05	7.24E+05	-0.83	procyanidin C2	Proanthocyanidins

pmp000275	3.14E+04	7.52E+04	-1.26	gambiriin A-1	Proanthocyanidins
pmp000273	2.57E+04	6.85E+04	-1.41	gambiriin B-3	Proanthocyanidins
mws0169	3.18E+06	9.00E+06	-1.50	Cyanidin 3-rutinoside	Anthocyanins
mws0171	8.18E+06	2.41E+07	-1.56	Cyanin chloride	Anthocyanins

Key metabolites are enclosed in red boxes.

Supplementary Table S4. Base information statistics.

Sample	Raw Data (bp)	Clean Data (bp)	AF_Q20 (%)	AF_Q30 (%)	AF_GC (%)
15 d ZK-1	7760782200	7728994925	98.05	94.30	47.53
15 d ZK-2	8149985700	8113066866	96.19	90.25	47.17
15 d ZK-3	7440591000	7408970072	98.03	94.29	47.34
45 d ZK-1	8391961800	8366095572	98.09	94.36	47.70
45 d ZK-2	7894026900	7867425490	98.03	94.20	47.62
45 d ZK-3	9351836400	9322501197	97.83	93.75	47.36
15 d N61-1	9682011900	9642763026	97.79	93.68	46.98
15 d N61-2	9845860500	9805718151	98.08	94.36	47.95
15 d N61-3	10474228800	10438291339	97.95	94.09	46.68
45 d N61-1	10721295300	10692850135	98.03	94.24	48.07
45 d N61-2	9989629800	9960436284	98.05	94.33	48.00
45 d N61-3	10472289300	10443240899	97.78	93.70	48.01

Supplementary Table S5. Pearson correlation coefficient between positively correlated metabolites and genes.

Gene Name	Gene ID	mws0997		mws1048		pmb0550		Annotation
		PPC	*P*-value	PPC	*P*-value	PPC	*P*-value	
CsLIMYBLX4	MSTRG.47184	0.90	5.20E-05	0.99	8.35E-10	0.98	6.21E-08	PREDICTED: L10-interacting MYB domain-containing protein-like isoform X4 [*Oryza brachyantha*]
CsMYB90	MSTRG.14584	0.80	1.79E-03	0.96	8.93E-07	0.92	2.18E-05	anthocyanin 1 [*Camellia sinensis*]
CsLIMYBL1	MSTRG.15849	0.91	3.47E-05	0.91	3.23E-05	0.93	1.58E-05	PREDICTED: L10-interacting MYB domain-containing protein-like [*Nicotiana attenuata*]
CsLIMYBL2	MSTRG.32788	0.85	4.29E-04	0.92	2.83E-05	0.91	5.03E-05	PREDICTED: L10-interacting MYB domain-containing protein-like, partial [*Setaria italica*]
CsMYB4	TEA_029924	0.83	8.87E-04	0.93	1.27E-05	0.91	3.11E-05	PREDICTED: myb-related protein Myb4 [*Theobroma cacao*]
CsBHLH018	MSTRG.29737	0.83	7.47E-04	0.96	9.91E-07	0.93	9.96E-06	transcription factor BHLH018, partial [*Vaccinium corymbosum*]
CsBHLH51	TEA_000397	0.71	9.97E-03	0.87	1.95E-04	0.86	3.91E-04	PREDICTED: transcription factor bHLH51 [*Vitis vinifera*]
CsBHLH49	MSTRG.19098	0.74	5.81E-03	0.82	1.21E-03	0.81	1.51E-03	PREDICTED: transcription factor bHLH49 isoform X2 [*Vitis vinifera*]
CsLIMYBLX2	MSTRG.45518	0.79	2.23E-03	0.82	1.16E-03	0.81	1.50E-03	PREDICTED: L10-interacting MYB domain-containing protein-like isoform X2 [*Glycine max*]
CsANS2	MSTRG.17031	0.67	1.82E-02	0.84	7.18E-04	0.79	2.46E-03	anthocyanidin synthase [*Camellia sinensis*]
CsANS3	TEA_014163	0.60	4.10E-02	0.78	2.98E-03	0.75	5.33E-03	anthocyanidin synthase [*Camellia sinensis*]
CsF3'H	TEA_014906	0.71	1.36E-02	0.83	7.86E-04	0.81	1.51E-03	flavonoid 3'-hydroxylase 1 [*Camellia sinensis*]
CsF3'H1	TEA_009006	0.61	1.99E-01	0.81	5.01E-02	0.78	6.96E-02	flavonoid 3'-hydroxylase 1 [*Camellia sinensis*]

PPC : Pearson correlation coefficient. mws0997, mws1048 and pmb0550 respectively represent the positively correlated metabolites petunidin 3-*O*-glucose, cyanidin 3-*O*-galactoside, and cyanidin 3-*O*-glucoside.

Supplementary Table S6. Pearson correlation coefficient between negatively correlated metabolites and genes.

Gene Name	Gene ID	mws1293		mws1295		mws1296		Annotation
		PPC	*P*-value	PPC	*P*-value	PPC	*P*-value	
CsLIMYBX3	TEA_001647	0.93	1.53E-05	0.96	5.66E-07	0.94	4.37E-06	L10-interacting MYB domain-containing protein-like isoform X3 [*Ananas comosus*]
CsBHLH25	TEA_014434	0.92	1.97E-05	0.93	8.65E-06	0.88	1.44E-04	PREDICTED: transcription factor bHLH18 [*Theobroma cacao*]
CsPPO4	TEA_004892	0.90	6.36E-05	0.91	3.39E-05	0.85	4.24E-04	polyphenoloxidase [*Camellia nitidissima*]
CsPPO5	TEA_024006	0.94	6.83E-06	0.94	7.80E-06	0.89	1.23E-04	polyphenoloxidase [*Camellia nitidissima*]
CsPPO6	TEA_022444	0.95	1.69E-06	0.97	2.51E-07	0.94	6.20E-06	polyphenoloxidase [*Camellia nitidissima*]

PPC : Pearson correlation coefficient. mws1293, mws1295, and mws1296 represent negatively correlated metabolites theaflavin, theaflavin-3'-gallate, and theaflavin-3, 3'-digallate, respectively.

Supplementary Table S7. Expression levels of genes with strong correlations with key metabolites in Zikui (ZK) and N61 tea plants at 15 d.

Gene Name	Gene ID	15 d N61 (mean-FPKM)	15 d ZK (mean-FPKM)	log_2FC (15 d ZK/15 d N61)	*P*-value	Regulated	Annotation
CsLIMYBLX4	MSTRG.47184	0.01	16.81	10.30	5.81E-34	up	PREDICTED: L10-interacting MYB domain-containing protein-like isoform X4 [*Oryza brachyantha*]
CsMYB90	MSTRG.14584	1.97	34.47	4.13	3.2E-113	up	anthocyanin 1 [*Camellia sinensis*]
CsLIMYBL1	MSTRG.15849	0.52	6.14	3.55	1.85E-32	up	PREDICTED: L10-interacting MYB domain-containing protein-like [*Nicotiana attenuata*]
CsLIMYBL2	MSTRG.32788	5.62	17.52	1.64	2.4E-35	up	PREDICTED: L10-interacting MYB domain-containing protein-like, partial [*Setaria italica*]
CsMYB4	TEA_029924	2.18	6.35	1.54	1.62E-12	up	PREDICTED: myb-related protein Myb4 [*Theobroma cacao*]
CsBHLH018	MSTRG.29737	0.00	2.11	11.05	3.04E-13	up	transcription factor BHLH018, partial [*Vaccinium corymbosum*]
CsBHLH51	TEA_000397	1.78	5.89	1.72	6.45E-09	up	PREDICTED: transcription factor bHLH51 [*Vitis vinifera*]
CsBHLH49	MSTRG.19098	1.57	3.14	1.00	9.39E-06	up	PREDICTED: transcription factor bHLH49 isoform X2 [*Vitis vinifera*]
CsLIMYBLX2	MSTRG.45518	0.59	3.78	2.68	8.47E-17	up	PREDICTED: L10-interacting MYB domain-containing protein-like isoform X2 [*Glycine max*]
CsANS2	MSTRG.17031	103.66	238.45	1.20	4.85E-22	up	anthocyanidin synthase [*Camellia sinensis*]
CsANS3	TEA_014163	10.84	18.50	0.77	1.13E-14	up	anthocyanidin synthase [*Camellia sinensis*]
CsF3'H	TEA_014906	0.51	1.05	1.04	0.00463	up	flavonoid 3'-hydroxylase 1 [*Camellia sinensis*]

CsF3'H1	TEA_009006	74.11	116.83	0.66	1.34E-12	up	flavonoid 3'-hydroxylase 3 [*Camellia sinensis*]
CsLIMYBX3	TEA_001647	13.13	0.03	-8.77	2.88E-22	down	L10-interacting MYB domain-containing protein-like isoform X3 [*Ananas comosus*]
CsBHLH25	TEA_014434	8.14	2.49	-1.71	7.58E-15	down	PREDICTED: transcription factor bHLH18 [*Theobroma cacao*]
CsPPO4	TEA_004892	42.29	5.01	-3.08	6.84E-65	down	polyphenoloxidase [*Camellia nitidissima*]
CsPPO5	TEA_024006	12.37	2.94	-2.07	5.27E-15	down	polyphenoloxidase [*Camellia nitidissima*]
CsPPO6	TEA_022444	14.79	1.64	-3.17	7.99E-17	down	polyphenoloxidase [*Camellia nitidissima*]
-	MSTRG.29079	0.03	3.57	6.74	6.13E-07	up	-
-	MSTRG.17415	0.54	5.15	3.25	1.17E-05	up	-
-	MSTRG.35246	3.03	8.56	1.50	5.02E-09	up	-
-	TEA_003607	7.63	32.39	2.09	4.91E-14	up	-
-	MSTRG.27856	0.24	5.65	4.56	1.09E-16	up	-
-	MSTRG.6132	0.06	4.20	6.21	1.19E-26	up	-
-	MSTRG.30086	0.08	1.28	4.06	0.007107	up	-
	MSTRG.31110	0.00	5.55	12.44	2.33E-17	up	-
-	MSTRG.2094	0.36	3.00	3.05	2.28E-07	up	-
-	MSTRG.12458	0.08	1.30	3.97	6.1E-05	up	-
-	MSTRG.33921	2.26	0.78	-1.54	2.55E-04	down	-
-	MSTRG.29824	0.94	0.08	-3.62	1.32E-02	down	-

-	MSTRG.14480	1.79	0.60	-1.57	1.34E-02	down	-
-	MSTRG.1185	2.65	0.51	-2.38	1.78E-03	down	-
-	MSTRG.38417	2.40	1.18	-1.03	2.50E-02	down	-
-	MSTRG.4356	5.79	2.05	-1.50	1.46E-07	down	-
-	MSTRG.46725	7.71	0.82	-3.24	9.07E-25	down	-
-	MSTRG.45508	0.97	0.42	-1.22	1.60E-01	down	-
-	TEA_019718	0.39	0.06	-2.63	5.69E-03	down	-

Supplementary Table S8. Expression levels of genes strongly related to key metabolites in Zikui (ZK) at 15 d and 45 d.

Gene Name	Gene ID	15 d ZK (mean-FPKM)	45 d ZK (mean-FPKM)	log_2FC(15 d ZK/45 d ZK)	*P*-value	Regulated	Annotation
CsLIMYBLX4	MSTRG.47184	16.81	3.10	2.44	1.19E-15	up	PREDICTED: L10-interacting MYB domain-containing protein-like isoform X4 [*Oryza brachyantha*]
CsMYB90	MSTRG.14584	34.47	0.16	7.75	2.81E-53	up	anthocyanin 1 [*Camellia sinensis*]
CsLIMYBL1	MSTRG.15849	6.14	3.32	0.89	9.46E-03	up	PREDICTED: L10-interacting MYB domain-containing protein-like [*Nicotiana attenuata*]
CsLIMYBL2	MSTRG.32788	17.52	10.46	0.74	4.81E-03	up	PREDICTED: L10-interacting MYB domain-containing protein-like, partial [*Setaria italica*]
CsMYB4	TEA_029924	6.35	0.69	3.21	2.55E-22	up	PREDICTED: myb-related protein Myb4 [*Theobroma cacao*]
CsBHLH018	MSTRG.29737	2.11	0.50	2.08	4.42E-05	up	transcription factor BHLH018, partial [*Vaccinium corymbosum*]
CsBHLH51	TEA_000397	5.89	0.52	3.51	7.33E-10	up	PREDICTED: transcription factor bHLH51 [*Vitis vinifera*]
CsBHLH49	MSTRG.19098	3.14	2.02	0.64	1.52E-01	up	PREDICTED: transcription factor bHLH49 isoform X2 [*Vitis vinifera*]
CsLIMYBLX2	MSTRG.45518	3.78	1.96	0.95	4.51E-03	up	PREDICTED: L10-interacting MYB domain-containing protein-like isoform X2 [*Glycine max*]
CsANS2	MSTRG.17031	238.45	97.25	1.29	1.83E-03	up	anthocyanidin synthase [*Camellia sinensis*]
CsANS3	TEA_014163	18.50	0.95	4.28	6.07E-62	up	anthocyanidin synthase [*Camellia sinensis*]
CsF3'H	TEA_014906	1.05	0.64	0.71	2.51E-01	up	flavonoid 3'-hydroxylase 1 [*Camellia sinensis*]
CsF3'H1	TEA_009006	116.83	28.42	2.04	3.76E-18	up	flavonoid 3'-hydroxylase 3 [*Camellia sinensis*]

CsLIMYBX3	TEA_001647	0.03	0.01	1.58	7.61E-01	up	L10-interacting MYB domain-containing protein-like isoform X3 [*Ananas comosus*]
CsBHLH25	TEA_014434	2.49	1.96	0.34	7.68E-01	up	PREDICTED: transcription factor bHLH18 [*Theobroma cacao*]
CsPPO4	TEA_004892	5.01	2.64	0.92	5.91E-03	up	polyphenoloxidase [*Camellia nitidissima*]
CsPPO5	TEA_024006	2.94	0.09	4.98	5.43E-19	up	polyphenoloxidase [*Camellia nitidissima*]
CsPPO6	TEA_022444	1.64	0.00	10.68	7.84E-06	up	polyphenoloxidase [*Camellia nitidissima*]
-	MSTRG.29079	3.57	0.32	3.50	2.34E03	up	-
-	MSTRG.17415	5.15	2.59	0.99	1.44E01	up	-
-	MSTRG.35246	8.56	3.70	1.21	9.01E03	up	-
-	TEA_003607	32.39	0.21	7.25	2.20E09	up	-
-	MSTRG.27856	5.65	2.30	1.30	2.79E02	up	-
-	MSTRG.6132	4.20	0.12	5.09	5.49E11	up	-
-	MSTRG.30086	1.28	0.09	3.83	3.50E02	up	-
	MSTRG.31110	5.55	3.03	0.87	2.46E02	up	-
-	MSTRG.2094	3.00	0.91	1.72	1.53E02	up	-
-	MSTRG.12458	1.30	0.00	10.35	3.06E05	up	-
-	MSTRG.33921	0.78	0.26	1.56	5.44E02	up	-
-	MSTRG.29824	0.08	0.00	6.26	5.64E01	up	-
-	MSTRG.14480	0.60	0.23	1.39	2.72E01	up	-
-	MSTRG.1185	0.51	0.00	8.99	1.47E02	up	-

-	MSTRG.38417	1.18	0.17	2.76	2.89E03	up	-
-	MSTRG.4356	2.05	0.57	1.85	1.78E02	up	-
-	MSTRG.46725	0.82	0.06	3.69	3.84E04	up	-
-	MSTRG.45508	0.42	0.00	8.70	3.74E03	up	-
-	TEA_019718	0.06	0.00	5.98	1.50E01	up	-

Supplementary Table S9. Pearson correlation coefficient between positively correlated metabolites and unannotated genes.

Gene Name	Gene ID	mws0997		mws1048		pmb0550		Annotation
		PPC	*P*-value	PPC	*P*-value	PPC	*P*-value	
-	MSTRG.29079	0.90	8.29E-05	0.91	4.01E-05	0.91	4.05E-05	-
-	MSTRG.17415	0.90	8.39E-05	0.87	2.60E-04	0.88	1.50E-04	-
-	MSTRG.35246	0.89	9.13E-05	0.88	1.39E-04	0.90	6.75E-05	-
-	TEA_003607	0.89	1.19E-04	0.90	5.51E-05	0.91	3.11E-05	-
-	MSTRG.27856	0.89	1.26E-04	0.86	3.65E-04	0.89	1.16E-04	-
-	MSTRG.6132	0.87	2.07E-04	0.87	2.40E-04	0.87	2.33E-04	-
-	MSTRG.30086	0.90	7.02E-05	0.92	2.08E-05	0.95	3.11E-06	-
-	MSTRG.31110	0.86	3.19E-04	0.91	4.05E-05	0.89	8.62E-05	-
-	MSTRG.2094	0.86	3.73E-04	0.84	5.65E-04	0.84	5.76E-04	-
-	MSTRG.12458	0.84	5.97E-04	0.89	1.28E-04	0.87	2.44E-04	-

PPC : Pearson correlation coefficient. mws0997, mws1048 and pmb0550 respectively represent the positively correlated metabolites petunidin 3-*O*-glucose, cyanidin 3-*O*-galactoside, and cyanidin 3-*O*-glucoside.

Supplementary Table S10. Pearson correlation coefficient between negatively correlated metabolites and unannotated genes.

Gene Name	Gene ID	mws1293		mws1295		mws1296		Annotation
		PPC	*P*-value	PPC	*P*-value	PPC	*P*-value	
-	MSTRG.33921	0.98	1.09E-08	0.97	7.80E-08	0.94	4.71E-06	-
-	MSTRG.29824	0.98	3.55E-08	0.99	2.21E-09	0.97	2.02E-07	-
-	MSTRG.14480	0.97	9.61E-08	0.97	3.40E-07	0.94	4.80E-06	-
-	MSTRG.1185	0.97	2.22E-07	0.97	7.66E-08	0.97	3.22E-07	-
-	MSTRG.38417	0.97	3.30E-07	0.93	1.03E-05	0.87	2.23E-04	-
-	MSTRG.4356	0.96	4.27E-07	0.96	5.04E-07	0.91	3.34E-05	-
-	MSTRG.46725	0.96	5.40E-07	0.98	4.68E-08	0.93	1.23E-05	-
-	MSTRG.45508	0.96	6.00E-07	0.92	1.74E-05	0.86	3.60E-04	-
-	TEA_019718	0.96	7.84E-07	0.98	2.00E-08	0.96	7.45E-07	-

PPC : Pearson correlation coefficient. mws1293, mws1295, and mws1296 represent negatively correlated metabolites theaflavin, theaflavin-3'-gallate, and theaflavin-3, 3'-digallate, respectively.

Supplementary Table S11. Expression levels of structural genes involved in anthocyanin biosynthesis in Zikui (ZK) at 15 d and 45 d.

Gene Name	Gene ID	15 d ZK (mean-FPKM)	45 d ZK (mean-FPKM)	log_2FC (15 d ZK /45 d ZK)	*P*-value	Regulated	Annotation
CsPAL1	TEA_003071	35.12	15.47	1.18	3.70E-04	up	phenylalanine ammonia-lyase [*Populus szechuanica*]
CsPAL2	TEA_020823	348.34	73.18	2.25	4.22E-07	up	phenylalanine ammonia-lyase [*Camellia japonica*]
CsPAL3	TEA_022055	101.93	18.54	2.46	3.69E-12	up	phenylalanine ammonia-lyase [*Camellia sinensis*]
CsPAL4	MSTRG.12851	66.50	17.34	1.94	5.20E-06	up	phenylalanine ammonia-lyase [*Camellia sinensis*]
CsC4H1	TEA_013313	551.85	191.38	1.53	2.60E-08	up	cinnamate 4-hydroxylase [*Osmanthus fragrans*]
CsC4H2	MSTRG.6357	351.34	67.81	2.37	1.00E-11	up	cinnamic acid 4-hydroxylase [*Camellia chekiangoleosa*]
CsC4H3	MSTRG.6358	5.34	1.52	1.82	1.41E-04	up	cinnamic acid 4-hydroxylase [*Camellia chekiangoleosa*]
Cs4CL1	TEA_023253	62.54	8.77	2.83	2.42E-32	up	PREDICTED: 4-coumarate--CoA ligase 2-like [*Jatropha curcas*]
Cs4CL2	MSTRG.2903	247.05	41.56	2.57	1.74E-16	up	4-coumaroyl CoA ligase [*Camellia sinensis*]
Cs4CL3	MSTRG.33696	2.11	0.00	11.05	7.37E-03	up	4-coumarate--CoA ligase 2 [*Glycine soja*]
CsF3'H2	TEA_006169	379.61	151.49	1.33	2.42E-03	up	flavonoid 3'-hydroxylase 2 [*Camellia sinensis*]
CsF3'H3	MSTRG.6382	21.65	0.69	4.98	2.01E-24	up	flavanone 3-hydroxylase, partial [*Camellia japonica*]
CsF3'5'H	TEA_011933	1239.64	428.30	1.53	1.21E-11	up	flavonoid 3',5'-hydroxylase [*Camellia sinensis* var. sinensis]
CsANS1	TEA_014156	1.15	0.05	4.43	1.05E-04	up	anthocyanidin synthase [*Camellia sinensis*]
CsANS2	MSTRG.17031	238.45	97.25	1.29	1.83E-03	up	anthocyanidin synthase [*Camellia sinensis*]
CsANS3	TEA_014163	18.50	0.95	4.28	6.07E-62	up	anthocyanidin synthase [*Camellia sinensis*]

CsUFGT	TEA_001487	70.11	1.94	5.17	3.22E-41	up	UDP-glycosyltransferase 78A15 [*Camellia sinensis*]

Supplementary Table S12. Primer sequences used in the study.

Gene Name	Gene ID	Forward Primer (5'→3')	Reverse Primer (5'→3')	Purpose
TUBA3	TEA_013193	TTAGGACAGGGACTTACCGC	TCCAAAAGCACAGCCACATC	qRT-PCR
CsLIMYBLX4	MSTRG.47184	ACCCGCAAGACCCACTAATC	GCCGTCAGAACCACCCAG	qRT-PCR
CsMYB90	MSTRG.14584	GGTCGTTGATTGCGGGTAGA	GGTAGTGGAAATGGCTCGCT	qRT-PCR
CsBHLH51	TEA_000397	TCGCATAAAAAGCAAGAGGCA	ACTGAGAACTAATCGCTGGAGG	qRT-PCR
CsANS2	MSTRG.17031	CTGCCCTCACCTTCATCCTC	CGCCCACGAAATCCTCACTT	qRT-PCR
CSF3'H1	TEA_009006	GGTTAGCAACAGCAGCCATC	TGGGGGAGAGAGCCGATTAG	qRT-PCR
CsLIMYBX3	TEA_001647	ATTTGGTGGTGGTTCGTCCT	TGAGGTTTGTGTTGAAGTTGCT	qRT-PCR
CsBHLH25	TEA_014434	CACTGTCGTTGCTCAGATGG	CACACTTCCTCCGCTCCTG	qRT-PCR
CsPPO4	TEA_004892	ACCACCATTCCCACCCATTC	AGCCTCCTAAGCCAATAAGCA	qRT-PCR
CsMYB90	MSTRG.14584	ATGGACAGTGTTTGCTGTG	TCATTCATCACCTAACAG	Gene cloning

Supplementary Figure legends:

Supplementary Figure S1: Kyoto Encyclopedia of Genes and Genomes (KEGG) enrichment of differential metabolites in Zikui (ZK) and N61 tea plants at 15 d.

Supplementary Figure S2: Gene Ontology (GO) enrichment of differentially expressed genes of Zikui (ZK) and N61 tea plants at 15 d and 45 d. (A) GO enrichment of 15 d N61 vs. 15 d ZK; (B) GO enrichment of 15 d ZK vs. 45 d ZK.

Supplementary Figure S3: KEGG enrichment of differentially expressed genes of Zikui (ZK) and N61 tea plants at 15 d and 45 d. The y-coordinate represents the pathway item, and the y-coordinate represents the rich factor. Dots represent the number of genes with significant differential expression, the size of the dot represents the number of genes with significant differential expression, where larger dots indicate more genes with significantly different expression. Dots in different colors represent different q-values.

Supplementary Figure S4: Expression level of *CsMYB90* in wild-type (WT) and transgenic *Nicotiana* tabacum 'K326' (n = 3 independent biological samples).

Supplementary Figure S5: Metabolomic data of Zikui (ZK) and N61 leaves. (A) PCA (Principal component analysis) clustering based on Zikui (ZK) and N61 metabolomic data; (B) HCA (hierarchical cluster analysis) clustering based on Zikui (ZK) and N61 metabolomic data.

Supplementary Figure S1

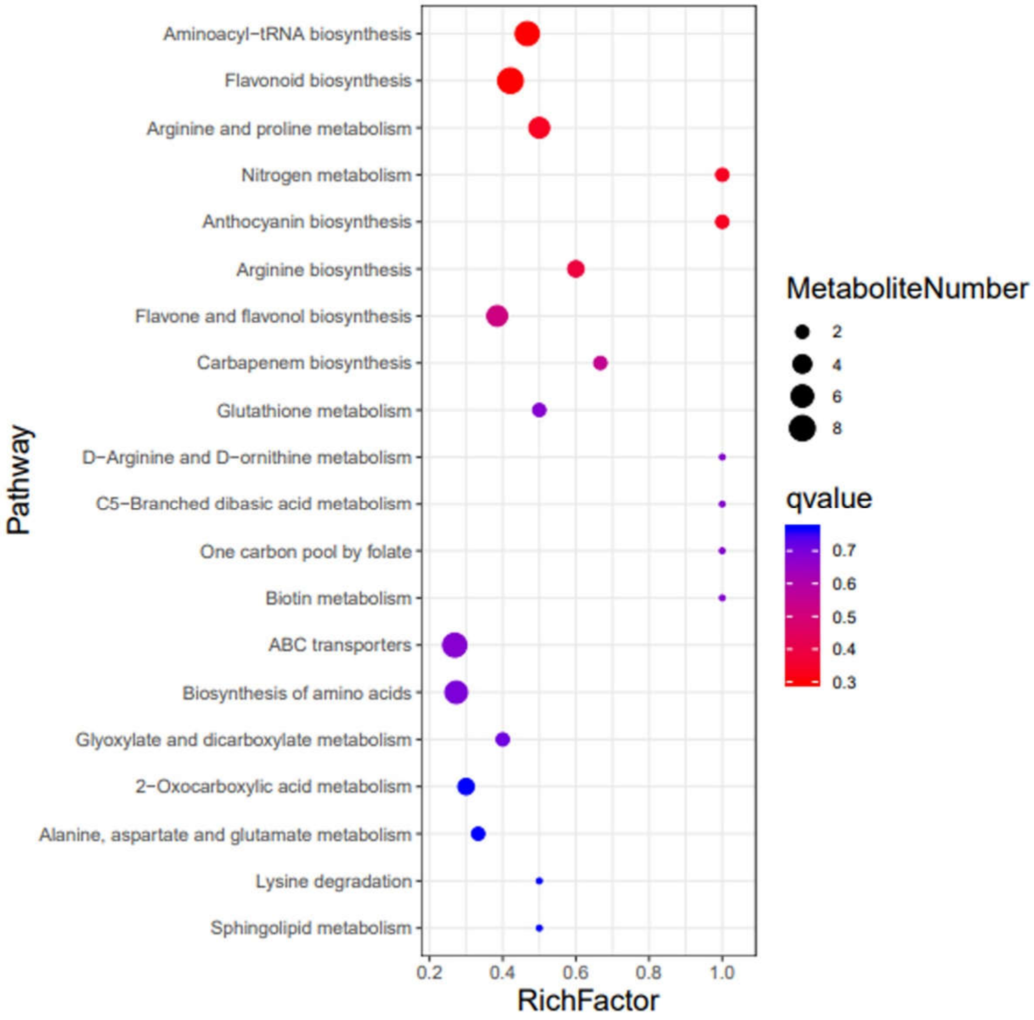

Supplementary Figure S2

A

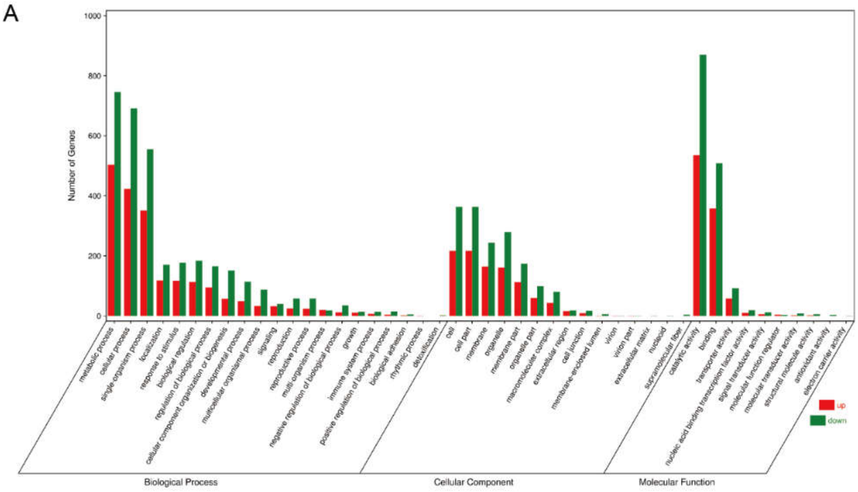

B

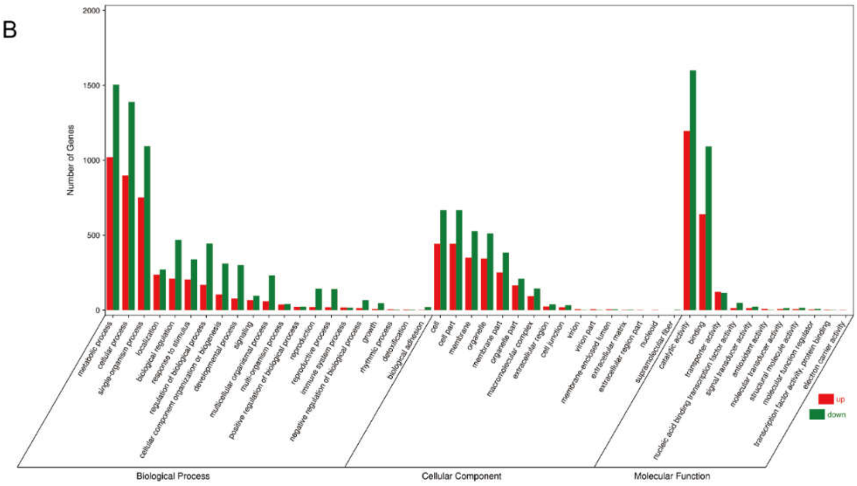

Supplementary Figure S3

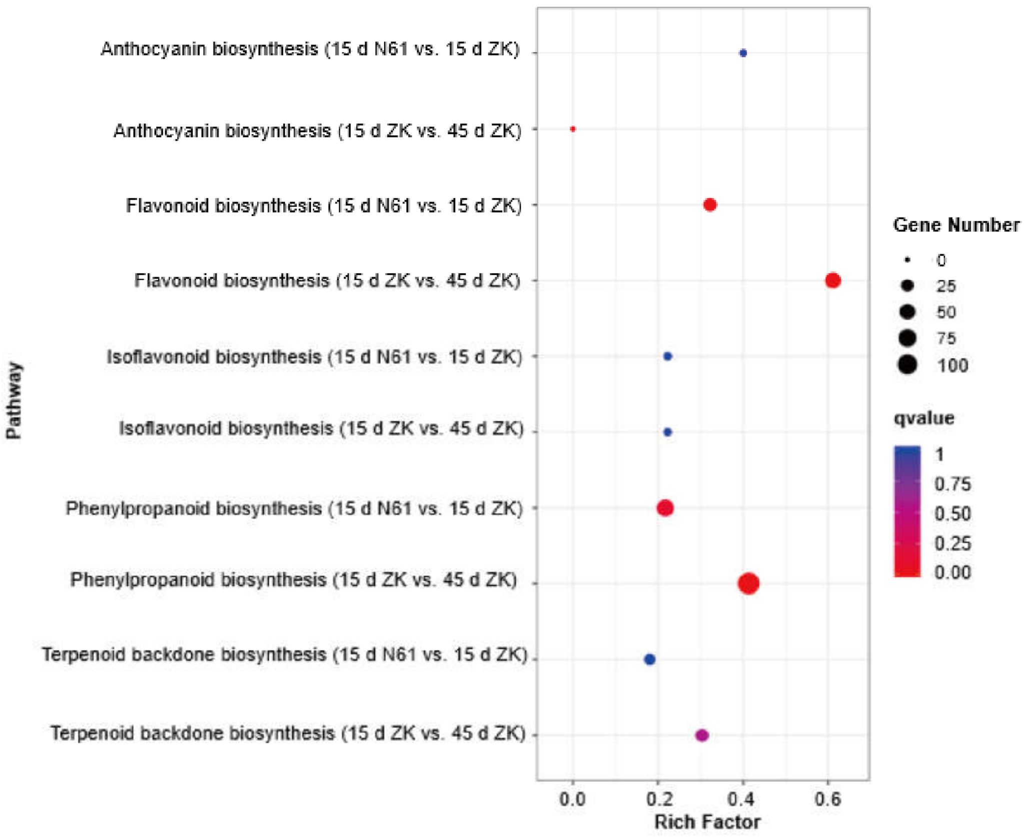

Supplementary Figure S4

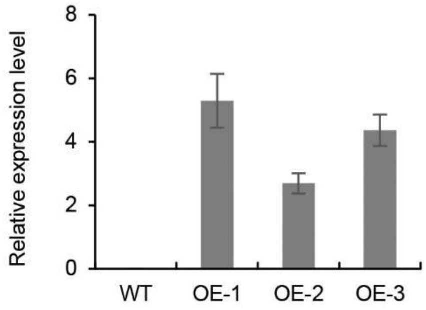

Supplementary Figure S5:

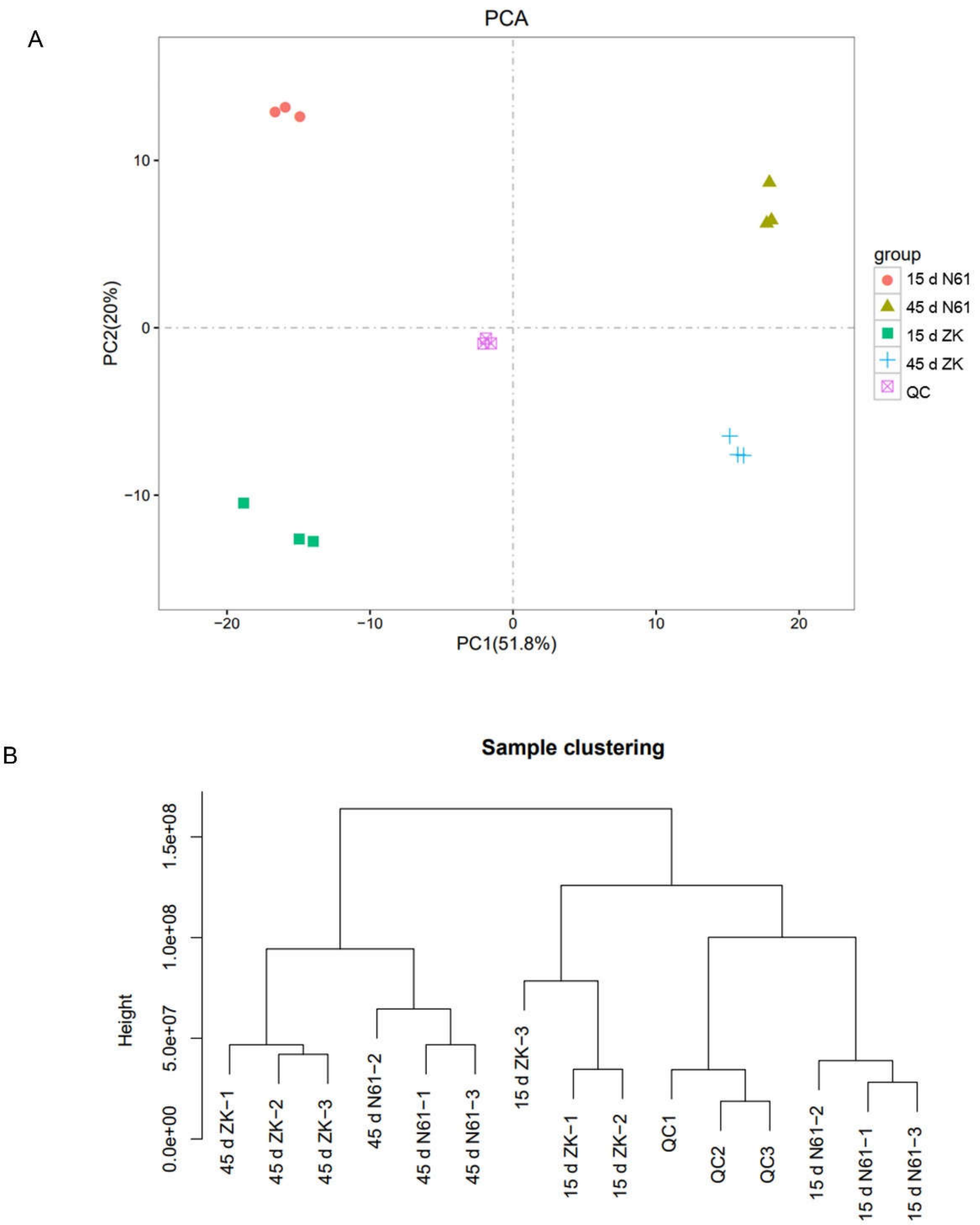

Supplement: Supplementary file 1 [file ijms-23-04780-s001.zip › supplementary-final.pdf]
